# Family Factors Affecting the Transition of Children from Normal Weight to Obesity in Mexico

**DOI:** 10.1089/chi.2021.0048

**Published:** 2022-02-25

**Authors:** Carlos Brambila-Paz, Domingo Faustino Hernandez-Angeles, Adan Silverio-Murillo, Abel Rodriguez-Tirado

**Affiliations:** School of Government, Tecnologico de Monterrey, Mexico City, México.

**Keywords:** family factors, Mexico, obesity, transition to obesity

## Abstract

***Background:*** This study is a longitudinal analysis of how the transition of a mother, father, or any other family member to obesity affects the likelihood of children 5–12 years of age becoming adolescents with overweight or obesity during the 7–10-year period between 2002 and the period from 2009 to 2012 in Mexico.

***Methods:*** The study used two rounds of the Mexican Family Life Survey, a multipurpose random national survey that collected information on 8441 households, including 38,233 individuals in 2002 and successfully followed up with 3202 children until the period from 2009 to 2012. We used logistic regressions to calculate how family characteristics related to the evolution of body mass indexes among children, controlling for individual, family weight-related characteristics, and the socioeconomic level of the family.

***Results:*** The transition of any family member toward obesity is more relevant in determining the transition to obesity among normal-weight children than socioeconomic level of the family and individual characteristics, such as sex, schooling, and occupation.

***Conclusions:*** The transition of any family member toward obesity is associated with the transition to obesity among normal-weight children. A family-based approach to obesity prevention has yet to be incorporated into national policies.

## Introduction

The latest Health and Nutrition survey (ENSANUT, 2018)^1^ stated that the combined prevalence of overweight and obesity among children 5–11 years of age in 2016 in Mexico was 35.6%. Among female children, the prevalence of overweight and obesity was 18.4% and 15.0%, respectively. Among male children, the prevalence of overweight and obesity was 17.7% and 20.1%, respectively. These abnormal weight levels in children are among the highest in the world.

Among adolescents 12–19 years of age, the combined prevalence of overweight and obesity in Mexico was 38.4% in 2018, 3.5% above the observed prevalence in 2012 (34.9%). Among teenage girls, the prevalence of overweight and obesity was 27.0% and 14.1%, respectively; among teenage boys, the prevalence of overweight and obesity was 20.7% and 15.1%, respectively. This trend is the most significant because nearly 39.0% of teenagers show an abnormal relation to weight/height by the age of 19. The health, social, and economic costs of such a trend remain to be determined. Most importantly, there is no clear sign of retrenchment of the obesity epidemic in Mexico.

The immediate cause of obesity is an excess accumulation of fat in the body due to an increased energy intake with respect to energy demand.^2–6^ However, such an imbalance between the energy value of food and energy expenditure is related to biological, social, cultural, political, local, and global economic factors. Such factors interact to affect the diet, the type of foods consumed, and the physical activity of individuals. Although these factors are indeed individual choices, they are mainly a family choice among children. To what extent do families affect the trends and tendencies of the obesity epidemic?

Several studies have investigated the extent to which adiposity, specifically body mass index (BMI), is passed down from one generation to the next.^7,8^ A comparison of health surveys from the United Kingdom, United States, China, Indonesia, Spain, and Mexico^7^ demonstrated that the intergenerational elasticity of BMI is comparable across time and countries, even controlling for the level of economic development of the country.

Drawing from family theories,^9–11^ it is possible to identify that family and parental relationships may affect an individual's propensity to become a person with overweight or obesity through several mechanisms, including genetic, cultural, psychological, behavioral, and social factors or, more likely, a combination of the above.

A biological family may impact an individual's propensity to become a person with overweight or obesity through genetic factors,^12,13^ even after controlling for environmental factors.^14^ The genetic factor has been identified in Mexico at the individual level,^15^ as well as in other groups and regions.^16^

Additionally, families may affect an individual's likelihood of becoming a person with obesity through cultural values and customs. Among several cultures, including that of Mexico, children with overweight are regarded as “healthy,”^17^ and there is social pressure concerning such standards.^18^ The main mechanisms through which such cultural factors operate are perceptions and attitudes.

There is substantive evidence concerning the misperceptions of parents,^19^ mothers in particular, regarding the weight of their children even when they are adolescents.^20,21^ Such misperceptions due to ignorance or underestimation of the health consequences of overweight or obesity increase the risks of their children developing obesity. Such a relationship has been observed even among poor indigenous populations in Mexico.^22,23^

The third mechanism through which families may affect children's weight is through the accepted level of activity^24^ and exercise,^25–27^ as well as diet.^28–30^ Family meal patterns have a major influence on overweight and obesity.^31^

There are several examples of how family meal patterns promote obesity, including snacking and the lack of exercise and physical activity. Parental influence on the propensity of children to developing obesity includes role modeling, lifestyle changes,^32–35^ preference for some types of food, calorie intake, and even genetic factors.^32–35^

When parents develop obesity, adolescents are also more likely to developing obesity,^36^ which is the topic that we address in this study.

This study provides robust longitudinal evidence that family weight-related characteristics are more relevant in determining the transition to obesity among normal-weight children than the socioeconomic level of the family and individual characteristics, such as sex, schooling, or occupation.

Although it is demonstrable that national programs in Mexico are very active and seek to reach the entire population, the obesity epidemic has not been controlled in the country. Partially, the limited impact of health policies is because the approach is still curative since there is not enough infrastructure or resources for prevention. Additionally, families and relatives are rarely incorporated in the treatment of overweight and obesity. A family-based approach to obesity prevention has not been incorporated into national policies.

## Materials/Subjects and Methods

We hypothesized that the transition to obesity is a family process that starts during childhood, wherein the transition of any family member toward obesity increases the likelihood that a child develops obesity over time.

### Data

We employed two rounds of the Mexican Family Life Survey (MxFLS)^37,38^ to estimate changes in the BMI of children 5–12 years of age in 2002, who were followed up with again in 2009–2012. This survey is a unique longitudinal study that enables the follow-up of a national random sample of individuals, households, and families containing extensive economic and demographic information at the household and individual levels.

The sample design ensured appropriate representation of rural and urban areas as well as four geographic regions in the country, following the official procedures of the National Institute of Statistics (INEGI). The sampling design of the surveys is probabilistic and therefore results may be generalized to the entire population of the country, which is predominantly poor. The survey design was multistage, stratified, and clustered, where the primary sampling unit is the household unit.

The MxFLS project is a multipurpose set of surveys, including economic, demographic, and social dynamics of rural and urban communities, households, families, and individuals, including migrants. The household questionnaire included the following variables: income and expenditures of the household and individual family members; decision-making concerning savings, credit, participation in public programs, education, and school attendance per household member; work histories; residence and coresidence; permanent and temporary migration; anthropometric and biological measures; and the use of health services, including pregnancies and reproductive health services as well as security issues.

The first round of MxFLS was conducted by INEGI, which is the official governmental agency responsible of collecting economic, social, and public health data in the country. With respect to informed consent, INEGI must comply with the Law of the National System of Statistical and Geographic Information, which establishes the requirement that all data collection should be subjected to informed consent and guaranteed confidentiality of information (articles 37 and 38). Additionally, INEGI has its own ethics board and is subject to external audits to guarantee compliance with the law.

The first round of the survey (MxFLS-1) was conducted in 2002 and included 8440 households (38,223 individuals) in 147 urban and rural communities throughout the country. The survey aimed to find and reinterview the same sample of informants and households from the MxFLS-1, including individuals who migrated within Mexico or immigrated to the United States, with a 90.0% success rate in recontacting and reinterviewing the family informants sample during the period from 2009 to 2012.^37,38^ Such high rate of recounting was possible because the baseline questionnaire included informed consent for follow-up and detailed information concerning alternative means of recontacting family members in the future. The endline survey took 3 years because it required follow-up of migrant families and individuals, even to the United States and several States within Mexico. It was necessary to conduct a snowball strategy to locate individuals who were separated from the family of origin.

MxFLS^37,38^ reports that both rounds used specialized and specifically trained personnel to conduct biometric measurements, including height and weight, along with other aspects, such as cephalic size, pregnancy status, hemoglobin count, and blood pressure. Fieldwork personnel were trained by doctors of the National Institute of Perinatology in Mexico to ensure the most accurate measurements of height using standard measuring tapes and reportedly high precision Taylor AO-11010-14 weight scales.

### Measures

Trained personnel accompanying each interviewer measured the weight and height of each of the family members.

Outcome variables: Change in BMI status between 2002 and the period from 2009 to 2012.

Change may be positive or negative, as shown in the results section. Given that BMIs change over time among children, the authors used the international reference of the World Health Organization (WHO) that sets Z-scores of weight/height for children and adolescents 5–12 years of age in 2002. The authors considered children with overweight as those who ranked between 1.00 and 2.00 Z-Scores as per the international standards; the authors considered children with obesity as those who measured more than 2.00 Z-Scores beyond international standards.

For adolescents and adults above 19.0 years of age, the BMI was calculated, and the participants were categorized into three groups: (1) low or normal: BMI ≥15 and BMI <25; (2) overweight: BMI ≥25 and BMI <30; and (3) obesity: BMI ≥30 and BMI ≤45.

Based on the 2002 and 2009–2012 measures of BMI, the authors constructed two dichotomic outcomes concerning BMI changes in children:

1. Low or normal BMI in 2002 and overweight BMI in 2009–2012. In this case, the comparison group included low- or normal-weight children who remained within the same standard over time.
2. Low, normal, or overweight BMI in 2002 and obesity BMI in 2009–2012. The comparison group in this case included low-, normal-, or children with overweight that did not transition to obesity over time.

These definitions aimed to evaluate the alternative paths to obesity.

### BMI/Weight Changes in Family Members

#### Individual characteristics

With respect to individual characteristics, the authors included sex, the highest level of education reached within the period of 2009–2012, whether the individual worked during the 12 months before the interview and whether the individual was working or studying at the time of endline visit.

### Family Weight-Related Variables

The questionnaire included a household roster in which the selected informant provided the demographic information of each household member, including age, date of birth, gender, type of relationship with the head of the household, marital status, level of education, employment, and occupation. Using the height/weight information, the authors were able to calculate the BMI of each family member and identified whether each individual was the head of the household, spouse, or other family member both in 2002 and the period from 2009 to 2012. In general, the household head is the father of the child, although not necessarily the biological father, as in the case of divorce and remarriage.

Drawing from the above information, the authors identified the following family weight-related variables for each individual:

1. At least two family members with overweight in 2002
2. At least two family members with obesity in 2002
3. The head of the household with obesity in 2002
4. The spouse with obesity in 2002
With respect to family changes in weight-related variables, the authors constructed two additional variables:
5. The head of the household developed obesity between 2002 and the period from 2009 to 2012
6. The spouse developed obesity between 2002 and the period from 2009 to 2012

To measure the relative socioeconomic level of individuals and households, the authors used the following five characteristics of the dwelling unit:

1. Number of rooms: dwelling unit with only one room and the family used the same room for cooking and sleeping.
2. Source of water: dwelling lacked piped water within the housing unit.
3. Sanitary service: dwelling unit lacked access to sewage treatment within the housing unit.
4. Trash collection: dwelling unit lacked a public collection of garbage and trash.
5. Fuel: dwelling unit used a fuel other than gas for cooking or heating.

The authors identified any housing unit that scored positive in one or more of the above indicators as precarious. The above measures were selected because they are applicable to rural and urban populations.

### Models

For each of the two possible outcomes, the authors fit logistic regression models with the baseline data as the reference category to estimate the odds ratios (ORs) for falling into the reference category.

The following is the form of the equation:


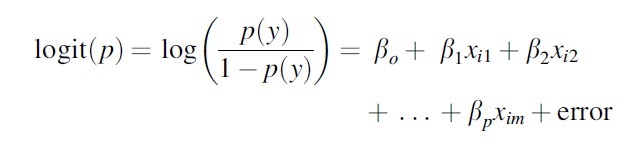


In the first model, *p* is the proportion of children transitioning from normal or underweight to overweight. In the second model, *p* is the proportion of children transitioning from underweight, normal weight, or overweight to obesity. Furthermore, *x_im_* refers to individual characteristics, including sex, educational level, work, and whether the individual was studying or working at the time of endline visit as control variables. Moreover, the authors included family weight-related variables, including the number of family members with overweight or obesity in 2002, if the head of the household or the spouse with obesity in 2002, and whether the head of the household or the spouse developed obesity between 2002 and the period from 2009 to 2012. Finally, the authors included a proxy variable for the socioeconomic level of the individual and household based on the conditions of the housing unit.

Girls who were pregnant in the 2009–2012 follow-up (4.0% of the sample) and those having extreme BMIs (1.6% with BMI <15; 0.4% with BMI >45) were excluded.

The study estimated models and coefficients with Stata 12 using the *logit* command (StataCorp 2020).

## Results

### BMI Changes in Children

Table 1 shows the unweighted distributions of the BMI changes in children, including the three BMI groups (low or normal weight, overweight, and obesity) in 2002 and 2009–2012. The results show that in 2002, 96.8% of children 5–12 years of age had a low or normal BMI (3101 out of 3202 children), and 3.1% had overweight or obesity. However, 7–10 years later, 23.9% and 9.0% had overweight and obesity, respectively, which means that nearly one third of the sample transitioned to an excess weight with respect to their height. The proportion of children with unhealthy BMI had a 10-fold increase over the analyzed period.

**Table 1.** Weight/Height Status of Children 5–12 Years of Age in 2002 and 2009–2012 in Mexico

	2009–2012 status^a^			
2002 status^a^	Underweight (%)	Overweight (%)	Obesity (%)	Total (%)
Underweight or normal weight (*n* = 3101)	68.85	23.48	7.67	100.00
Overweight (*n* = 87)	12.64	34.48	52.87	100.00
Obesity (*n* = 14)	21.43	50.00	28.57	100.00
Total (*n* = 3202)	67.11	23.89	8.99	100.00

^a^ For children and adolescents 5–18 years of age (months 61–227): (1) Underweight is _zbfa ≤−2; (2) Normal weight/height is (_zbfa ≥2.1 and _zbfa ≤1.0); (3) Overweight is (_zbfa ≥1.1 & _zbfa ≤2.0), and (4) Obesity is (_zbfa >2.0). _zbfa are WHO Z-Scores by age and sex. For young adults, 19 years of age or older: (1) low or normal: BMI ≥15 and BMI <25, (2) overweight: BMI ≥25 and BMI <30, (3) obese: BMI ≥30 and BMI ≤45.

Source: Calculations using the MxFLS of 2002 and 2009–2012.

BMI, body mass index; MxFLS, Mexican Family Life Surveys; WHO, World Health Organization.

Table 2 shows the sample sizes without adjustments for each of the comparison groups. Of the 3101 children who had low or normal weight/high Z-scores in 2002, 23.5% and 7.7% became affected by overweight and obesity, respectively, with a total of 31.2% transitioning to abnormal weight (group 1). Group 2 included 3188 cases that had low, normal, or overweight Z-scores in 2002, of which 8.9% developed obesity by the period from 2009 to 2012.

**Table 2.** Descriptive Statistics of Response, Independent, and Control Variables

Variable	Proportion (%)	95% CI
Low or normal BMI in 2002 and overweight in 2009–2012 (*n* = 3101)	31.20	29.50–32.80
Low, normal, or overweight in 2002 and obese in 2009–2012 (*n* = 3188)	8.90	7.90–9.90
Individual characteristics 2009–2012		
Male (*n* = 3199)	51.30	49.60–53.00
Single (*n* = 3175)	93.40	92.50–94.20
Work past 12 months (*n* = 3198)	18.00	16.70–19.30
Primary (*n* = 2180)	10.40	9.10–11.70
Secondary (*n* = 2180)	40.90	38.80–42.90
Not studying or working (*n* = 3198)	22.50	21.00–23.90
Family variables		
At least two family members with low BMI in 2002 (*n* = 3202)	53.50	51.80–55.30
At least two family members with normal BMI in 2002 (*n* = 3202)	63.90	62.20–65.50
At least two family members with overweight BMI in 2002 (*n* = 3202)	64.40	62.80–66.10
At least two family members obese BMI in 2002 (*n* = 3202)	45.40	43.70–47.10
Head of household obese in 2002 (*n* = 3202)	60.90	59.20–62.60
Spouse obese in 2002 (*n* = 3202)	62.20	60.50–63.90
Other members obese in 2002 (*n* = 3202)	47.80	46.10–49.50
Head of household transition to obesity (*n* = 3202)	8.60	7.60–9.50
Spouse transition to obesity (*n* = 3202)	9.00	8.00–10.00
At least one family member BMI transition to obese (*n* = 3202)	58.60	56.90–60.30
Socioeconomic proxy in 2002		
Precarious housing unit (*n* = 3202)	61.10	59.50–62.80

Source: Calculations using the MxFLS of 2002 and 2009–2012.

CI, confidence interval.

Table 2 shows the descriptive statistics of the control variables without weights to expand the sample to the national level. Furthermore, the table displays the family characteristics related to weight: 64.4% of the studied families had at least two members with overweight in 2002, and 45.4% had at least two members with obesity. In 60.9% of the households, the head of the household had obesity, and in 62.2%, the spouse had obesity at the starting point. During the observation period, 8.6% of household heads developed obesity and 9.0% of wives became persons with obesity; however, in 58.6% of households, at least one family member changed the BMI status to obesity.

### Family Factors Affecting the Transition to Obesity

Table 3 shows that families with at least two members with obesity are 4.886 times (95% confidence interval [CI] = 4.700–5.078) more likely to have children undergoing the transition from low or normal weight to overweight. Along with this process, the second most significant factor is the spouse becoming a person with obesity over the 7–10-year period (OR = 3.022, 95% CI = 2.981–3.065).

**Table 3.** Odds Ratios That Children with Low or Normal Weight/Height in 2002 Transition to Overweight in 2009–2012 and Odds Ratios that Children with Low, Normal, or Overweight in 2002 Transition to Obesity in 2009–2012, Mexico (*N* = 3202)

	Low or normal in 2002 and overweight or obese in 2009–2012				Low, normal, or overweight in 2002 to obese in 2009–2012			
	OR	Robust standard error	*p* > |z|	95% CI	OR	Robust standard error	*p* > |z|	95% CI
Male	0.872	0.003	0.000	0.866–0.879	0.484	0.003	0.000	0.479–0.490
Work	1.140	0.013	0.000	1.115–1.165	0.518	0.008	0.000	0.504–0.534
Primary school	1.237	0.006	0.000	1.225–1.248	0.586	0.005	0.000	0.577–0.596
Secondary school	1.062	0.004	0.000	1.054–1.071	0.634	0.004	0.000	0.626–0.641
Dropout	0.478	0.002	0.000	0.473–0.483	3.259	0.036	0.000	3.189–3.330
Not studying or working	0.844	0.009	0.000	0.826–0.863	0.581	0.008	0.000	0.565–0.598
At least two family members underweight	0.572	0.002	0.000	0.568–0.576	0.355	0.002	0.000	0.351–0.358
At least two family members normal weight	0.848	0.003	0.000	0.841–0.855	0.596	0.004	0.000	0.589–0.604
At least two family members overweight	1.315	0.007	0.000	1.302–1.328	0.519	0.004	0.000	0.510–0.527
At least two family members obese	4.886	0.096	0.000	4.700–5.078	0.582	0.012	0.000	0.560–0.606
Head of household obese 2002	1.451	0.007	0.000	1.438–1.465	1.572	0.012	0.000	1.548–1.597
Spouse obese 2002	1.745	0.008	0.000	1.729–1.761	1.143	0.009	0.000	1.126–1.159
Other family members obese 2002	0.277	0.006	0.000	0.267–0.288	2.607	0.053	0.000	2.505–2.713
Head of household transition to obesity in 2009–2012	1.643	0.011	0.000	1.622–1.664	0.384	0.007	0.000	0.371–0.397
Spouse transition to obesity in 2009–2012	3.022	0.021	0.000	2.981–3.065	0.504	0.010	0.000	0.485–0.524
Precarious housing unit	1.630	0.006	0.000	1.619–1.642	1.214	0.007	0.000	1.201–1.227

Source: Calculations using the MxFLS of 2002 and 2009–2012.

OR, odds ratio.

Families wherein the spouse is a person with obesity at the starting point are 1.745 times (95% CI = 1.729–1.761) more likely to have their children transitioning from low or normal weight to overweight or obesity. However, it is not only relevant whether the head of the household (in general the husband) had obesity at the starting point (OR = 1.451, 95% CI = 1.438–1.465) but also if he had transitioned to obesity over the study period (OR = 1.643, 95% CI = 1.622–1.664).

With respect to individual characteristics, probably the most significant result of this study is that individual characteristics are less significant than family factors on the chances of developing overweight or obesity. Male children are less likely to transition to obesity (OR = 0.872, 95% CI = 0.866–0.879) than female children. Adolescents (in 2009) who finished only primary school are more likely to transition to obesity (OR = 1.237, 95% CI = 1.225–1.248) than adolescents that continue or finish secondary school (OR = 1.062, 95% CI = 1.054–1.071). Work during the previous 12 months to the endline survey slightly increased the odds of developing obesity (OR = 1.140, 95% CI = 1.115–1.165) compared with adolescents who did not work.

The right-hand panel of Table 3 illustrates that weight-related family factors are a significant antecedent to obesity: in 2002, children in families with at least two family members with obesity were 2.607 times more likely (95% CI = 2.505–2.713) to transition to obesity than families without members with obesity.

## Discussion

The presence of other family members with obesity increases the likelihood of a normal-weight child developing overweight or obesity. A father or a mother with obesity increased the likelihood of a child transitioning to overweight or obesity over the 7–10-year analyzed period. In general, the transition of any family member toward obesity is more relevant in determining the transition to obesity among normal-weight children than the socioeconomic level of the family and individual characteristics, such as sex, schooling, or occupation.

These results indicate that the process of developing obesity is largely a family issue, including a variety of mechanisms, such as sharing a diet type, feeding habits within the family, similar economic conditions, and reproduction of cultural stereotypes. The results also show that typically the process of developing obesity starts when another family member developing obesity or if the head of the household or the spouse develops obesity; this was noted even after controlling for individual factors.

The transition to obesity depends on eating habits, the level of activity, and genetic and epigenetic factors. Since the last decade of the 20th century, changes in traditional food culture, the high availability and accessibility of industrialized foods with high energy density, increased consumption of sugary drinks, and the mass marketing of processed foods have affected the immediate causes of obesity.

The results presented demonstrate that, even in the context of an increased availability of processed foods, family factors are also important as individual factors such as age, sex, schooling, and occupation. These results indicate that the process of developing obesity is largely a family issue. When the head of the household or the spouse develop obesity, the chances of developing overweight or obesity increase substantially for children. The results also show that often times, the process of developing obesity starts when another family member transitions also into obesity or if the head of the household or the spouse develop obesity; this was noted even after controlling for individual factors.

One limitation of the available information is that the data collection was restricted to up to three household visits, such that only the teenagers who were present at the time of the survey had their measurements taken. In 2002, MxFLS-1 collected BMI information on 7029 children 5–12 years of age. The 2009–2012 survey (MxFLS-3) successfully collected the weight/height information of 5438 adolescents 12–22 years of age from the original sample, with a 22.6% attrition rate. However, the models used in this study included the weight-related characteristics of all family members over time. Considering that weight/height measures were applied for present family members only (including up to three home visits), the number of cases with complete family information were 3202 children. Therefore, the sample analyzed included 3202 children 5–12 years of age in 2002 and who remained living with the family of origin until 2009–2012.

Although the attrition rate is acceptable in a 7–10-year longitudinal study, we acknowledge, as a possible limitation of the study, the lack of evidence concerning trends among missing youths in the sample although we restricted the analysis to children that remain within the household of origin.

Although it is demonstrable that several national programs in Mexico and elsewhere are very active and seek to reach entire populations; however, the obesity epidemic has not been controlled even in developed countries, in particular among children. Partially, the ineffectiveness or public policies is because the approach at large is still curative: families and relatives are rarely incorporated in the treatment of overweight and obesity, as has been endorsed by national and international agencies.

First, prevention requires multidisciplinary health teams that work in conjunction with an integral approach, including diet, physical activity, behavioral therapy, support groups, active patient participation, and alternative sources of clean water. Such an approach should be directed to families and not only individuals, as recommended by the WHO.

Second, the focus of the programs remains reactive, in the sense that service providers make recommendations when they have the first contact with the patient in health centers, clinics, or hospitals. In practice, health providers do not follow-up individual cases or families over time to assess progress in the treatment of overweight or obesity or to promote prevention among other children in the family. This suggests the need to adopt a reach-out approach aiming to identify and assist housing units and families at risk. This is a family-based approach to preventive medicine.

Third, it is necessary to address the role of parents and older adults to prevent children from gaining weight.

The previous recommendations require modifications to the current models in primary care. Even if an excellent service is achieved through the services that consider the doctor as the main service provider, such actions would be insufficient. It is necessary to promote cultural and social changes and modernize the organization of services. These changes must include the active participation of individuals, families, and communities with the support of multidisciplinary teams.

## Authors' Contributions

D.F.H.-A. contributed to the main idea and purpose of the study and conducted the preliminary analysis to develop the base model for populations 19 years of age and older. C.B.-P. adapted the base model for teenagers and conducted the analysis and drafted the article. A.S.M. provided methodological guidance to specify the models analyzed and to conduct a robust analysis. A.R. conducted the literature review and developed the conceptual framework of the study. All authors interpreted the results and their implications, commented on the articles at all stages, and approved the final submitted version.

## References

[1] Shamah-Levy T, Vielma-Orozco E, Heredia-Hernández O, et al. Encuesta Nacional de Salud y Nutrición 2018–19: Resultados Nacionales. Cuernavaca, México: Instituto Nacional de Salud Pública, 2020.

[2] Astrup A, Dyerberg J, Selleck M, Stender S. Nutrition transition and its relationship to the development of obesity and related chronic diseases. Obes Rev 2008;9:48–52. 18307699 10.1111/j.1467-789X.2007.00438.x

[3] Bouchard C. Gene**–**environment interactions in the etiology of obesity: Defining the fundamentals. Obesity (Silver Spring) 2008;16:S5–S10. 19037213 10.1038/oby.2008.528

[4] Bray GA. Etiology and pathogenesis of obesity. Clin Cornerstone 1999;2:1–15. 10.1016/s1098-3597(99)90001-7 10696281

[5] Gardner C. The etiology of obesity. Mod Med 2003;100:242–247. 12847865

[6] Weinsier RL, Hunter GR, Heini AF, et al. The etiology of obesity: Relative contribution of metabolic factors, diet, and physical activity. Am J Med 1998;105:145–150. 9727822 10.1016/s0002-9343(98)00190-9

[7] Dolton P, Xiao M. The intergenerational transmission of body mass index across countries. Econ Hum Biol 2017;24:140–152. 27987491 10.1016/j.ehb.2016.11.005

[8] Fernández-Paredes F, Sumano-Avendaño E. [Obesity in childhood and adolescence: Risk factors]. Bol Med Hosp Infant Mex 1986;43:53–56. 3954858

[9] Bell D. *Exploring Family Theories*, 4th ed. J Fam Theory Rev 2018;10:308–312.

[10] Fair C, Rogliano M, Byrne L, et al. The influence of family systems theory obesity treatment on adolescents' and parents' perspectives of family dynamics. J Adolesc Health 2018;62:S57.

[11] Haselschwerdt ML. Family theories: Foundations and applications. J Fam Theory Rev 2018;10:692–697.

[12] Goodarzi MO. Genetics of obesity: What genetic association studies have taught us about the biology of obesity and its complications. Lancet Diabetes Endocrinol 2018;6:223–236. 28919064 10.1016/S2213-8587(17)30200-0

[13] Campbell Am LV. Genetics of obesity. Aust Fam Physician 2017;46:456–459. 28697287

[14] Sheikh AB, Nasrullah A, Haq S, et al. The interplay of genetics and environmental factors in the development of obesity. Cureus 2017;9:e1435. 28924523 10.7759/cureus.1435 PMC5587406

[15] Dávila-Torres J, González-Izquierdo JJ, Barrera-Cruz A. [Obesity in Mexico]. Rev Med Inst Mex Seguro Soc 2015;53:240–249. 25760754

[16] Dávila-Rodriguez MI, Cortés-Gutiérrez EI, Rivera-Prieto RA, et al. [Epidemiological genetics of obesity in Northeast Mexico. Ascertainment of nuclear informative families]. *Gac Med Mex* 2005;141:243–246. 16025994

[17] Agne AA, Daubert R, Munoz ML, et al. The cultural context of obesity: Exploring perceptions of obesity and weight loss among Latina immigrants. J Immigr Minor Health 2012;14:1063–1070. 22130571 10.1007/s10903-011-9557-3 PMC3818086

[18] Baquero B, Molina M, Elder J, et al. Neighborhoods, social and cultural correlates of obesity risk among Latinos living on the U.S.–Mexico border in Southern California. J Health Care Poor Underserved 2016;27:700–721. 27180704 10.1353/hpu.2016.0063

[19] McLaughlin EA, Campos-Melady M, Smith JE, et al. The role of familism in weight loss treatment for Mexican American women. J Health Psychol 2017;22:1510–1523. 26929169 10.1177/1359105316630134

[20] Vallejo M, Cortes-Rodríguez BA, Colin-Ramirez E. Maternal underestimation of child's weight status and health behaviors as risk factors for overweight in children. J Pediatr Nurs 2015;30:e29–e33. 10.1016/j.pedn.2015.02.009 25764943

[21] Vázquez-Velázquez V, Kaufer-Horwitz M, Méndez JP, et al. Eating behavior and psychological profile: Associations between daughters with distinct eating disorders and their mothers. BMC Womens Health 2017;17:74. 28874196 10.1186/s12905-017-0430-y PMC5585917

[22] Arrizabalaga-Amarelo R, Mendieta-Zerón H. Obesity among parents and children from an indigenous rural community in Mexico. Sao Paulo Med J 2007;125:370–371.1831761110.1590/s1516-31802007000600014

[23] Herrera-Huerta EV, García-Montalvo EA, Méndez-Bolaina E, et al. [Overweight and obesity in indigenous nahuas from Ixtaczoquitlan, Veracruz, Mexico]. Rev Peru Med Exp Salud Publica 2012;29:345–349. 23085795

[24] Brouwer SI, Küpers LK, Kors L, et al. Parental physical activity is associated with objectively measured physical activity in young children in a sex-specific manner: The GECKO Drenthe cohort. BMC Public Health 2018;18:1033. 30126399 10.1186/s12889-018-5883-x PMC6102934

[25] Kunin-Batson AS, Seburg EM, Crain AL, et al. Household factors, family behavior patterns, and adherence to dietary and physical activity guidelines among children at risk for obesity. J Nutr Educ Behav 2015;47:206–215. 25748634 10.1016/j.jneb.2015.01.002 PMC4428928

[26] Van Allen J, Borner KB, Gayes LA, Steele RG. Weighing physical activity: The impact of a family-based group lifestyle intervention for pediatric obesity on participants' physical activity. J Pediatr Psychol 2015;40:193–202. 25241402 10.1093/jpepsy/jsu077

[27] Evans GW, Jones-Rounds ML, Belojevic G, Vermeylen F. Family income and childhood obesity in eight European cities: The mediating roles of neighborhood characteristics and physical activity. Soc Sci Med 2012;75:477–481. 22595070 10.1016/j.socscimed.2012.03.037

[28] Barr-Anderson DJ, Adams-Wynn AWA, DiSantis KI, Kumanyika S. Family-focused physical activity, diet and obesity interventions in African-American girls: A systematic review. Obes Rev 2013;14:29–51. 10.1111/j.1467-789X.2012.01043.x PMC3524349 23057473

[29] Braden A, Strong D, Crow S, Boutelle K. Parent changes in diet, physical activity, and behavior in family-based treatment for childhood obesity. Clin Pediatr (Phila) 2015;54:494–497. 24928574 10.1177/0009922814538702 PMC5588888

[30] Liu J, Hay J, Faught BE, et al. Family eating and activity habits, diet quality and pre-adolescent overweight and obesity. Public Health 2012;126:532–534. 22560409 10.1016/j.puhe.2012.02.012

[31] Berge JM, Miller J, Watts A, et al. Intergenerational transmission of family meal patterns from adolescence to parenthood: Longitudinal associations with parents' dietary intake, weight-related behaviours and psychosocial well-being. Public Health Nutr 2018;21:299–308. 29037275 10.1017/S1368980017002270 PMC5947321

[32] Balantekin KN, Birch LL, Savage JS. Family, friend, and media factors are associated with patterns of weight-control behavior among adolescent girls. Eat Weight Disord 2018;23:215–223. 28315233 10.1007/s40519-016-0359-4 PMC5601019

[33] Bates CR, Buscemi J, Nicholson LM, et al. Links between the organization of the family home environment and child obesity: A systematic review. Obes Rev 2018;19:716–727. 29520946 10.1111/obr.12662

[34] Sigmund E, Sigmundová D, Badura P, Madarasová Gecková A. Health-related parental indicators and their association with healthy weight and overweight/obese children's physical activity. BMC Public Health 2018;18:676. 29855285 10.1186/s12889-018-5582-7 PMC5984306

[35] Ma Z, Hample D. Modeling parental influence on teenagers' food consumption: An analysis using the Family Life, Activity, Sun, Health, and Eating (FLASHE) Survey. J Nutr Educ Behav 2018;50:1005–1014. 30414664 10.1016/j.jneb.2018.07.005

[36] Ajslev TA, Ängquist L, Silventoinen K, et al. Stable intergenerational associations of childhood overweight during the development of the obesity epidemic. Obesity (Silver Spring) 2015;23:1279–1287. 25959297 10.1002/oby.21060

[37] Rubalcava L, Teruel G. User's guide for the Mexican Family Life Survey First Wave. In: Hogares (ed), Primera Encuesta Nacional sobre Niveles de Vida de los Hogares. ENSAV-l. Mexico: CIDE-UIA, 2006.

[38] Rubalcava L, Teruel G. User's guide for the Mexican Family Life Survey Third Wave. Mexico: CIDE-UIA, 2013.

